# Optimizing Care for Ugandans with Untreated Abdominal Surgical Conditions

**DOI:** 10.5334/aogh.2427

**Published:** 2019-04-01

**Authors:** Elissa K. Butler, Tu M. Tran, Anthony T. Fuller, Christine Muhumuza, Sarah Williams, Joao R. N. Vissoci, Samuel Luboga, Michael M. Haglund, Fredrick Makumbi, Moses Galukande, Jeffrey G. Chipman

**Affiliations:** 1 University of Washington Department of Surgery, Seattle, WA, US; 2 Duke University Division of Global Neurosurgery and Neurology, Durham, NC, US; 3 University of Minnesota Medical School, Minneapolis, MN, US; 4 Duke University School of Medicine, Durham, NC, US; 5 Makerere University School of Public Health, Kampala, UG; 6 Duke Global Health Institute, Durham, NC, US; 7 Makerere University College of Health Sciences Department of Anatomy, Kampala, UG; 8 Duke University Department of Neurosurgery, Durham, NC, US; 9 Makerere University College of Health Sciences, Kampala, UG; 10 University of Minnesota Department of Surgery, Minneapolis, MN, US

## Abstract

**Background:**

Abdominal operations account for a majority of surgical volume in low-income countries, yet population-level prevalence data on surgically treatable abdominal conditions are scarce.

**Objective:**

In this study, our objective was to quantify the burden of surgically treatable abdominal conditions in Uganda.

**Methods:**

In 2014, we administered a two-stage cluster-randomized Surgeons OverSeas Assessment of Surgical Need survey to 4,248 individuals in 105 randomly selected clusters (representing the national population of Uganda).

**Findings:**

Of the 4,248 respondents, 185 reported at least one surgically treatable abdominal condition in their lifetime, giving an estimated lifetime prevalence of 3.7% (95% CI: 3.0 to 4.6%). Of those 185 respondents, 76 reported an untreated condition, giving an untreated prevalence of 1.7% (95% CI: 1.3 to 2.3%). Obstructed labor (52.9%) accounted for most of the 238 abdominal conditions reported and was untreated in only 5.6% of reported conditions. In contrast, 73.3% of reported abdominal masses were untreated.

**Conclusions:**

Individuals in Uganda with nonobstetric abdominal surgical conditions are disproportionately undertreated. Major health system investments in obstetric surgical capacity have been beneficial, but our data suggest that further investments should aim at matching overall surgical care capacity with surgical need, rather than focusing on a single operation for obstructed labor.

## Background

Abdominal operations account for at least two-thirds of surgical volume in low-income countries, and cesarean sections, specifically, account for 30%. Five billion people around the world lack access to timely, affordable surgical care [1]. Given the success of political advocacy and the commitment of health system leaders, cesarean section is now the most widely available major operative procedure [2]. In Uganda, a first-referral hospital’s surgical output is predominantly limited to cesarean sections, herniorrhaphies, and emergency laparotomies [3]. But capacity is challenged by a shortage of surgical providers and lack of infrastructure to support safe surgery [4,5,6].

The College of Surgeons of East, Central, and Southern Africa (COSECSA) has proposed task-shifting essential surgical procedures to trained nonphysician providers—citing decades of positive experience training clinical officers to perform emergency cesarean sections, particularly in Uganda [7]. In the third edition of *Disease Control Priorities*, global surgery experts recommended a package of essential surgical procedures to be delivered at the level of a first-referral hospital, of which 10 of 18 address abdominal conditions [8]. However, further efforts are needed to improve *overall* surgical capacity; currently, except for certain prioritized operations such as cesarean sections, most individuals do not have access to high-quality surgical care. Fortunately, the infrastructure that has been established for pregnancy-related operations can also efficiently support other major operations, thereby helping to address unmet surgical need.

In Uganda today, existing evidence of surgical capacity is available only at the facility level. Less is known about surgical need, especially at the population level. Facility-based evidence overlooks individuals who are unable to access care; moreover, only 11.2% of Ugandans live within 5 km of mini-hospitals known as Health Centre IVs (which are one level below first-referral hospitals) [9]. A more accurate account of disease burden requires data on the underlying population, including individuals who have not sought care at a health care facility. Therefore, we performed a cross-sectional household survey to quantify disease burden, using a nationally representative sample. By demonstrating the need for nonobstetric abdominal surgical operations, we envisage that, in the future, general surgical services might receive a fairer share of governmental and donor resources [10].

## Methods

### Survey Instrument

For this study, we slightly modified the Surgeons OverSeas Assessment of Surgical Need (SOSAS) survey, adding more response options but maintaining comparability with previous national SOSAS studies [11,12,13]. For example, we added an image of the abdomen with nine sections, so respondents could identify where their abdominal pain was localized. Also in the Abdomen portion of the survey, we included an expanded list of signs and symptoms, such as dyspepsia, blood in vomit or stool, and jaundiced eyes.

To administer the survey, we partnered with the Performance Monitoring and Evaluation 2020 Uganda program (which administers a nationwide, longitudinal cluster-randomized population survey on family planning, for which resident enumerators originate from, or have close familial ties to, their enumeration area and are fluent in both English and the predominant language spoken in the area). For our survey, we used the same resident enumerators and the same enumeration areas, as described previously [14]. At each household, the head of household responded to the enumerator’s questions on demographics, health facility access, and details of any household deaths within the previous 12 months. In addition, two randomly selected household members responded to a head-to-toe verbal interview in which they self-reported any existing or previous surgically treatable conditions. Because surgically treatable conditions of the abdomen have common and often nonspecific signs and symptoms, enumerators were instructed to document each condition, as described by the respondent, in an open response section.

### Study Setting

Uganda is a country of 34.9 million inhabitants [15], and 8.5% of the government’s budget is allocated to the health sector [16]. Health Centre IVs are the lowest-level health facilities capable of performing major operations, but only on an emergent basis. Availability of cesarean sections is used as a leading indicator of Health Centre IV surgical capacity. The first-referral hospitals are general hospitals and district hospitals.

### Sampling and Data Collection

To capture a nationally representative sample, we used a cluster sample designed by the Uganda Bureau of Statistics that selected 105 enumeration areas, based on probability proportional to size sampling from 80,000 enumeration areas stratified by type (urban or rural) and by 10 subregions [17]. To calculate our study’s sample size, we used 1% precision and 6.3% prevalence proportion of unmet surgical need, inclusive of all surgically treatable conditions, per our pilot study in a single district in Uganda [18].

On-site data collection occurred between August 20, 2014 and September 12, 2014. In each enumeration area, the enumerators randomly selected households from a sampling frame (which had been created six months earlier by fully listing and mapping the area). Individuals of all ages were eligible to be respondents; those who could not respond for themselves were assisted by a surrogate. Both the household and individual components of the SOSAS survey were administered in the language preferred by the respondents. The data were recorded in English using Open Data Kit mobile phone software (University of Washington, Seattle, WA, USA).

### Data Analysis

To determine whether an abdominal condition was treated, required further treatment, or was not treated but would have been surgically treatable, surgeons and medical students rated each condition. To do so, they assessed the structured and open response portions of the survey for each abdominal condition. They were blinded to one another’s ratings. Raters discussed any discrepancies in rating until they reached a consensus. We excluded from our analysis any condition or cause of death that we deemed not likely to have been surgically treatable.

With support from the Uganda Bureau of Statistics, we computed household-level, individual-level, and post-stratification weights to determine weighted proportions, confidence intervals (CIs), and regression model outputs through Taylor series linearization for variance estimation. For all statistical analyses, we used Stata 13 (StataCorp, College Station, TX, USA). We set statistical significance at *p* < 0.05. To compare proportions, we used chi-squared tests. To create a map of surgically treatable abdominal conditions, we used QGIS (Free and Open Source Software).

### Ethics

This study was approved by the Uganda Council of Science and Technology, the Makerere University School of Medicine research and ethics committee, the Duke University Health System institutional review board, and the University of Minnesota institutional review board. Before administering the survey, each head of household and the two randomly selected household members provided verbal informed consent. Children through the age of 7 years participated with parental consent; children ages 8 to 17 provided assent in addition to parental consent. For adults who were not competent to provide consent, a surrogate family member provided consent.

## Results

We enumerated 4,248 individuals in 2,315 households (97.1% response rate). Of those respondents, 185 reported at least one surgically treatable abdominal condition in their lifetime, giving a lifetime prevalence of 3.7% (95% CI: 3.0 to 4.6%). Of those 185 respondents, 76 reported an untreated condition, giving an untreated prevalence of 1.7% (95% CI: 1.3 to 2.3%), which extrapolates to 597,202 (95% CI: 442,702 to 804,366) individuals with an untreated abdominal surgical condition. Of all respondents with an untreated surgical condition, 16.2% (95% CI: 12.2 to 20.2) had an untreated abdominal condition.

**Figure 1:**
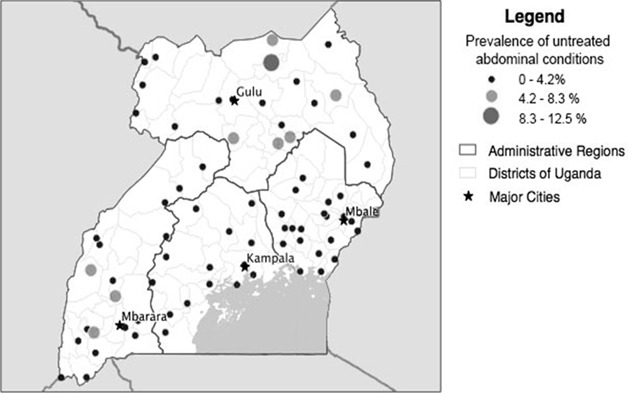
Prevalence of untreated abdominal surgical conditions by enumeration area (n = 105).

**Figure 2:**
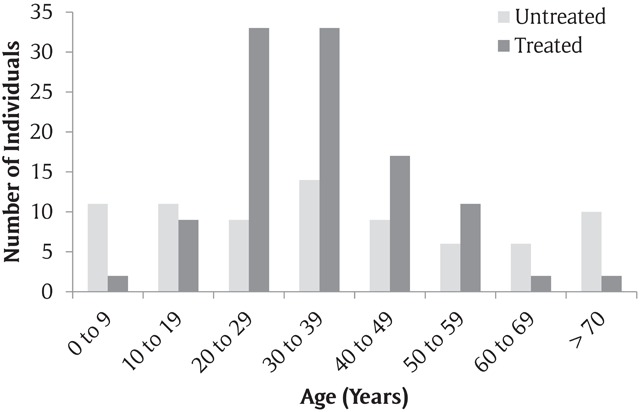
Age distribution of respondents with treated and untreated abdominal surgical conditions.

The Northern, Karamoja, and Southwest subregions of Uganda had the highest prevalence of untreated abdominal surgical conditions (Figure 1). The mean age of respondents with an abdominal surgical condition was 35.2 years (standard deviation [SD]: 20.1). Respondents who were 19 years or younger and 60 years or older had a higher proportion of untreated abdominal surgical conditions, as compared with the overall proportion of untreated abdominal surgical conditions (Table 1, Figure 2). Male respondents and respondents with no education also had a higher proportion of untreated abdominal surgical conditions (Table 1).

**Table 1:** Characteristics of respondents with at least one surgically treatable abdominal condition in their lifetime (*n* = 185).

	Full cohort (*n* = 185)*	Treated (*n* = 109)	Untreated (*n* = 76)	Proportion untreated, %

**Age, years, n (%)**				

0–19	33 (17.8)	11 (10.1)	22 (29.0)	66.7**
20–39	89 (48.1)	66 (60.6)	23 (30.3)	25.8**
40–59	43 (23.2)	28 (25.7)	15 (19.7)	34.9
≥60	20 (10.8)	4 (3.7)	16 (21.0)	80.0**
**Gender, n (%)**				

Female	133 (71.9)	88 (80.7)	45 (59.2)	33.8
Male	52 (28.1)	21 (19.3)	31 (40.8)	59.6**
**Enumeration area type, n (%)**				

Rural	147 (79.5)	86 (78.9)	61 (80.3)	41.5
Urban	38 (20.5)	23 (21.1)	15 (19.7)	39.5
**Education level, n (%)**				

None	80 (43.2)	34 (31.2)	46 (60.5)	57.5**
Primary	59 (31.9)	40 (36.7)	19 (25.0)	32.2
≥ Secondary	46 (24.9)	45 (41.3)	11 (14.5)	23.9**

* 238 lifetime abdominal conditions among 185 respondents. ** Significantly different from overall proportion of respondents with untreated abdominal conditions (41.1%).

Respondents reported a total of 238 abdominal conditions; obstructed labor (52.9%) accounted for most of them. We noted a large difference in treatment status between respondents with obstructed labor and respondents with all other types of conditions (Table 2). Only 5.6% of reported cases of obstructed labor were untreated; in contrast, 73.3% of reported abdominal masses were untreated. Conditions that began within the month before administration of the survey were more likely to be untreated.

**Table 2:** Reported abdominal surgical conditions (*n* = 238).

	All abdominal conditions (*n* = 238)*	Treated (*n* = 161)	Untreated (*n* = 77)	Proportion untreated, %

**Type of condition, n (%)**				

Obstructed labor	126 (52.9)	119 (73.9)	7 (9.1)	5.6**
Mass	30 (12.6)	8 (5.0)	22 (28.6)	73.3**
Pain	26 (10.9)	8 (5.0)	18 (23.4)	69.2**
Hernia	25 (10.5)	12 (7.5)	13 (16.9)	52.0**
Wound	15 (6.3)	9 (5.6)	6 (7.8)	40.0
Other	16 (6.7)	5 (3.1)	11 (14.3)	68.8**
**Timing of onset, n (%)**			

<1 month	15 (6.3)	6 (3.7)	9 (11.7)	60.0**
1–12 months	37 (15.5)	19 (11.8)	18 (23.4)	48.6
>12 months	186 (78.2)	136 (84.5)	50 (64.9)	26.9

* 238 lifetime abdominal conditions among 185 respondents. ** Significantly different from overall proportion of untreated abdominal conditions (32.4%).

In assessing health care-seeking behaviors and barriers to care, we found that most respondents (98.8%) with treated abdominal surgical conditions had sought formal health care. In contrast, only 74.0% of respondents with untreated abdominal surgical conditions had sought formal health care (Table 3). Among respondents with untreated abdominal surgical conditions, 20.8% had sought traditional healers, as compared to 6.8% of respondents with treated abdominal surgical conditions. Cesarean section was the most frequently performed surgical procedure (51.7%) for respondents with abdominal surgical conditions (Table 3), followed by other major operations (14.4%) and by minor operations (12.0%).

**Table 3:** Health care–seeking behaviors among respondents with abdominal surgical conditions (*n* = 238).

	All abdominal conditions (*n* = 238)*	Treated (*n* = 161)	Untreated (*n* = 77)

**Traditional healer sought, n (%)**	27 (11.3)	11 (6.8)	16 (20.8)
**Formal health care sought, n (%)**	216 (90.8)	159 (98.8)	57 (74.0)
Type of care received, n (%)**			

None	25 (11.6)	1 (0.6)	24 (42.1)
Cesarean section	123 (51.7)	119 (74.8)	4 (7.0)
Other major procedure	31 (14.4)	23 (14.5)	8 (14.0)
Minor procedure	26 (12.0)	16 (10.1)	10 (17.5)
Referral to higher level of care	11 (5.1)	0 (0)	11 (19.3)
**Reason for no care, n (%)^†^**			

Lack of money	13 (52.0)	–	13 (54.2)
Perceived lack of need	7 (28.0)	1 (100.0)	6 (25.0)
Lack of transport	3 (12.0)	–	3 (12.5)
Fear or lack of trust	3 (12.0)	–	3 (12.5)
Other	3 (12.0)	–	3 (12.5)
**Reason for not seeking care, n (%)^‡^**			

Lack of money	12 (54.5)	–	12 (60.0)
Lack of transport	6 (27.3)	–	6 (30.0)
Perceived lack of need	5 (22.7)	2 (100.0)	3 (15.0)
Fear or lack of trust	5 (22.7)	–	5 (25.0)
Lack of social support	5 (22.7)	–	5 (25.0)
Other	2 (9.1)	–	2 (10.0)

* 238 lifetime abdominal conditions among 185 respondents. ** Among conditions where individual decided to seek health care (n = 216). ^†^ Among conditions where individuals sought health care but care was not received (n = 25). Multiple responses allowed. ^‡^ Among conditions in which individuals did not seek health care (n = 22). Multiple responses allowed.

Among the 25 respondents who sought care but did not receive any, the most common reasons were lack of money (52.0%), followed by perceived lack of need for surgical care (28.0%). Among the 22 respondents who did not seek care, the most common reasons were lack of money (54.5%) and lack of transport (27.3%).

Of the 153 reported deaths, 14 were due to abdominal surgical conditions (weighted %: 8.9%, 95% CI: 3.5 to14.4%). With regard to household surgical deaths, the most frequently cited anatomic region was the abdomen (weighted %: 26.1%, 95% CI: 12.7 to 39.6). Abdominal pain was the most frequently cited symptom immediately before death (35.7%), followed by abdominal distention (21.4%) and acquired deformity (21.4%). No deaths due to untreated obstructed labor or other complications of pregnancy were reported. Children and adolescents (ages 0 to 19 years) accounted for 50% of abdominal surgical deaths (Table 4).

**Table 4:** Deaths due to abdominal surgical conditions (*n* = 14).

Symptoms before death, n (%)	

Abdominal pain	5 (35.7)
Abdominal distention	3 (21.4)
Acquired deformity	3 (21.4)
Mass	2 (14.3)
Wound (due to injury)	1 (7.2)
**Age, years, n (%)**	

0–19	7 (50.0)
20–39	3 (21.4)
40–59	2 (14.3)
≥60	2 (14.3)
**Gender, n (%)**	

Female	8 (57.1)
Male	6 (42.9)

## Discussion

This study is the first nationally representative, population-based quantification of abdominal surgical need in Uganda. We found that Ugandans with nonobstetric abdominal surgical conditions are disproportionately undertreated. About 50% of major operations at government hospitals in Uganda are cesarean sections [4], and yet the need for operations to treat obstructed labor has still not been completely met. A much larger problem in Uganda is the unmet need for general surgery operations. Even at tertiary-referral hospitals, which perform a higher proportion of nonobstetric operations, cesarean sections account for nearly half of all major operations [4]. In low- and middle-income countries, efforts to improve access to cesarean sections have significantly decreased the unmet need for operations to treat obstructed labor. As seen in our study, only 6% of individuals with obstructed labor go untreated, as compared with 32% of individuals with other abdominal conditions. Thus, governments need to build upon existing surgical infrastructure to improve overall surgical capacity. Nonobstetric abdominal surgical conditions warrant a higher proportion of governmental and donor resources.

According to our survey, children and adolescents in Uganda had a higher proportion (66.7%) of untreated abdominal conditions than young and middle-aged adults; moreover, of Ugandans who died of an abdominal surgical condition, half were children and adolescents. Programs that focus on improving child health, such as the Uganda Newborn Study, should be expanded to include surgical education, thereby helping enhance parental knowledge of pediatric surgical conditions [19]. A similar community-based approach should be taken for older adults (those who are at least 60), since we found a high proportion of untreated abdominal conditions (80.0%) in this age group. In 2009, the Ministry of Gender, Labour, and Social Development published the National Policy for Older Persons, prioritizing health improvement for older individuals. Future policies should include education on and treatment of surgical conditions [20].

Given our survey results, we extrapolated that about 600,000 Ugandans currently have untreated abdominal surgical conditions. Of those 600,000 individuals, about 25% may not be able to obtain a surgical consultation because of lack of funds or transport. Nearly 75% of our survey respondents had sought medical care but had not received the needed operation. When surgically treatable conditions worsen, so do the financial costs to individuals and societies [21,22].

We found that among Ugandans the main barrier to seeking care for an abdominal condition was lack of financial resources. Increased health expenditures and the successful future rollout of the planned National Health Insurance Scheme will be key to improving access to surgical care in Uganda. Investing in elective surgical services is cost-effective and promotes economic growth [22]. General and obstetric surgery delivered at a first-referral hospital has already been shown to be cost-effective, with some studies suggesting the cost to be as low as 5% of the gross domestic product (GDP) per capita in low-income countries [23,24,25,26,27]. Those studies mainly included acute abdominal presentation, obstetric emergencies, and orthopedic trauma. But in a recent cost-effectiveness analysis of a private hospital in India, Chatterjee et al. showed that, even with inclusion of elective general surgery, cost levels would remain only slightly above 10% of the GDP per capita [28].

The cost-effectiveness of surgical care should appeal to stakeholders, but service availability is only part of the problem. Poor health literacy also contributes to unmet surgical need. Results from population-based studies like ours challenge current approaches, which focus on increasing surgical capacity. Certainly, an inadequate surgical workforce, subpar equipment, and disorganized infrastructure severely limit progress, but governments must also focus on improving health literacy. Among many of our survey respondents, we noted a perceived lack of need for surgery. Lower levels of educational attainment and the seeking of traditional healers were significantly associated with the presence of untreated abdominal surgical conditions. Simply removing the financial barriers to elective surgery will not be mirrored by an increased demand for elective surgery [29,30]. Reducing the burden of disease through elective surgery depends on individuals, of their own volition, seeking surgical care in a timely manner. Sensitization at the community level will be required to enhance knowledge of common surgically treatable conditions.

Other studies have found that individuals sought traditional healers (instead of formal health care) because of a generalized mistrust of the safety and effectiveness of surgery [29,30,31]. In Sierra Leone, a household survey of individuals older than 50 found that 42% sought a traditional healer for their surgically treatable condition [32]. Sociocultural factors are known to significantly influence health care-seeking behaviors among individuals with non-surgical diseases, and those same factors likely influence the perceived need for surgical care [29,30,31,33]. As elective surgery becomes more readily available in low- and middle-income countries, simultaneous efforts are essential to educate the community about the safety and effectiveness of surgical care.

Another recent study demonstrated that advocacy for surgical services is stymied by poor framing of the issue and by lack of specific solutions beyond calls for major investments in health system resources, such as surgical providers [34]. In light of the increasing evidence in favor of task-shifting for some essential operations [35], we recommend a gradual increase in training surgical providers. As Health Centre IVs, general hospitals, and district hospitals expand the availability of cesarean sections, they are also increasingly equipped to perform other major abdominal operations for nonobstetric patients. By investing in training a robust surgical workforce, general surgery operations could be performed in higher volume at all such facilities. At some of these facilities, medical officers (general practitioners with medical school and one year of internship training) are currently performing as many as 1,400 operations per year, most of which are cesarean sections [36]. Continuing medical education programs must be more robustly implemented, so that medical officers (with high operative demands) are more effectively mentored. One way to achieve this goal is to train medical officers at surgical camps, transferring skills at an intensive pace [37].

This study has limitations that should be addressed in future epidemiologic studies of surgical diseases, especially in the abdominal region, where signs and symptoms do not easily yield a diagnosis without hospital-based diagnostic tools. Reliance on self-reporting for abdominal surgical diseases is complicated: some symptoms might be clearly perceived and medically manageable; other conditions have no or ambiguous symptoms. Our projection of 600,000 Ugandans with untreated abdominal surgical conditions could be either an overestimation or an underestimation. Linden et al. [38] showed that household surgical surveys are prone to low sensitivity, especially concerning conditions in the groin region. Gupta et al. [13] validated the SOSAS instrument in Nepal, finding an acceptable 94.6% alignment between the self-reporting by respondents and visual examination by clinicians.

Finally, an added limitation to our study’s design was the need for surgeons to review survey enumerators’ work, in order to rate the likelihood of surgical amenability of a condition. Naturally, some information is lost during on-site interviews with respondents, and some is lost during the interpretation and rating process. Such loss is particularly true for abdominal conditions. But for the interviews, we attempted to achieve high fidelity through our modifications to the original SOSAS instrument.

Moving forward, more data are needed from longitudinal epidemiologic surveys and from routine health facility reports that focus on surgical diseases and surgical outcomes. Governments in low- and middle-income countries must expand overall surgical capacity (not just access to cesarean sections) and must increase the ability of community outreach programs to improve health literacy on surgically treatable conditions.
